# Trust, Participation, and Power: Community Perspectives from the Equity in Public Health Initiative

**DOI:** 10.1007/s10900-026-01612-8

**Published:** 2026-07-23

**Authors:** Shaniece Criss, Roger S. Abim-Karmon, Jody Teel, Vanessa Rodríguez, Adia Bennon, Sally Wills, Sarah Gonzales, Dalmondeh D. Nayreau, Vaishnavi Bharadwaj, Hannah G. Kim, Jennifer Woollery, Melissa L. Fair

**Affiliations:** 1 Department of Health Sciences, Furman University, 3300 Poinsett Hwy, Greenville, SC 29613 USA; 2 Institute for the Advancement of Community Health, Furman University, 3300 Poinsett Hwy, Greenville, SC 29613 USA; 3 LiveWell Greenville, 770 Pelham Rd, SC 29615 Greenville, USA

**Keywords:** Community power, Trust, Community participation, Equity, Local government

## Abstract

Health disparities arise from unequal power relations shaping resource allocation, governance, and community agency. Community power is a key mechanism for addressing disparities. This study examines how visible, hidden, and invisible power operate within a U.S. county to inform inclusive governance. We conducted seven focus groups with Black and Hispanic residents (*n* = 68) and sixteen semi-structured interviews with elected officials, government staff, and advocacy leaders in Greenville County, South Carolina. Data were collected between March and December 2024. Transcripts were collaboratively coded in NVivo and analyzed using thematic analysis guided by Heller et al.’s Three Faces of Power framework. Visible power appeared through inequitable resource distribution, limited transparency, and barriers to civic participation. Hidden power functioned through faith based organizations, schools, nonprofits, and social media, connecting residents with decision makers. Invisible power reflected how socioeconomic status, race and ethnicity, immigration, and language shaped perceptions of agency and trust. Participants called for participatory structures that move beyond consultation toward shared authority and sustained engagement. Strengthening community power through structural reforms, inclusive governance practices, and targeted civic engagement may improve health outcomes and reduce disparities. Future research should examine scalable co-governance models.

## Introduction

### The Importance of Community Power in Addressing Health Inequities

Community power is a fundamental tool for addressing health inequities and remodeling set systems that perpetuate disparities [1, 2]. Community power is “the ability of communities most impacted by structural inequity to develop, sustain, and grow an organized base of people who act together through democratic structures to set agendas, shift public discourse, influence who makes decisions, and cultivate ongoing relationships of mutual accountability with decision makers that change systems and advance health equity” [3]. Fostering community power within public health involves empowering those most affected by health inequities to lead efforts in improving their own health outcomes [3–5]. Research has shown that health inequities are not random but stem from the unequal allocation of power and resources, shaped by policies and other mechanisms, which manifest in unequal social, economic, and environmental conditions that contribute to intergroup differences in health outcomes both within and between societies [6–8]. Power itself has been studied and posited over time as a fundamental cause and determinant of health inequity, given the ability it has to create, maintain, and widen social inequalities through ongoing dynamic struggles and because of the political nature of health [9–11].

Hence, the importance of community power resides in its capacity to confront and transform the structural obstacles that give rise to health inequalities. These methods extend beyond conventional top-down health interventions (i.e., centralized expert-driven decision-making that tends to exclude the community) by directly involving community members in determining needs, formulating plans, and holding decision-makers accountable [2, 4, 12]. One strategy that researchers have proposed for intervening in existing power dynamics to foster health justice include a two-pronged process of building power among populations harmed by health inequity while breaking power for those who perpetuate health disparities [10]. Strategies to build power including community organizing, coalition-building and social movements, and institutional negotiations, and strategies to break power include profit minimization and administrative regulation and enforcement [10]. Additionally, McCartney et al. also argues that health inequalities persist and are reproduced because of unequal power relations [9]. The authors proposed a framework which operationalizes power and can be used in policy-making and public health practice to address health inequalities [9]. The multidimensional framework seeks to identify the sources of power (e.g., economic power, state power), the spaces where power operates (e.g., media environments, labor markets), the positions holding power (e.g., politicians, academics), and the forms of power (e.g., agenda-setting, shaping norms & beliefs) [9].Through analyzing different issues with this framework, such as understanding the underlying power relations, the actors who benefit, and what institutions are involved, we can work towards creating structural or institutional changes through solutions that will promote health.

### The Social Determinants of Health and the Political Determinants of Health

Two important categorical determinants of health support our understanding of power and its relationship to health inequities. The Social Determinants of Health (SDOH) are the everyday conditions that shape people’s ability to live healthy lives, including access to safe housing and neighborhoods, reliable transportation, healthy food, quality healthcare, education and employment [13]. When people lack suitable conditions and are faced with issues such as language barriers at the clinic or long commutes to buy groceries, their health suffers not because of personal choices, but because of systems that make well-being harder to reach. Addressing the social determinants of health means creating environments where people feel safe, respected, and connected, and they are not just treated when they are sick, but supported in staying well.

Another factor that may promote or inhibit health is the Political Determinants of Health (PDOH) which Dawes et al. defines as the legislative, judicial, and executive actions that allocate resources and power, thereby instigating the social determinants of health [14]. These political determinants are not merely background factors, but rather they are intentional political decisions made through systematic processes that actively influence health outcomes [14]. Importantly, these factors go beyond personal policy decisions and include a complicated web of institutional, commercial, and governmental interests that essentially shape the environments in which people live, work, and receive healthcare. Essentially, the political determinants of health operationalize power through policy decisions that impacts health.

Iton et al. makes a compelling case that breaking down these structural barriers requires communities to build real political power so they can influence the decisions that happen at the top [2]. This idea resonates throughout recent research which points to the same truth; that is, lasting change comes when people get involved in civic life, organize at the grassroots level, and have a genuine say in how their communities are governed [5, 15]. This change is about reforming structures that have traditionally silenced underrepresented voices. Public health transforms from a service into a shared obligation based on justice and fairness when communities are empowered to speak up for themselves, shape policy, and hold institutions accountable in meaningful ways, using civic participation as a mechanism to influence PDOH.

### The Role of Civic Engagement, Trust, and Participation in Shaping Equitable Systems

Civic engagement, trust, and participation are powerful mechanisms for transforming health systems and challenging deeply entrenched power dynamics. The critical link between these power dynamics and health lies in how structural inequities create invisible barriers that limit community agency, directly impacting individuals’ ability to access healthcare, make health decisions, and create supportive living environments. Research increasingly shows that when communities feel connected and invested in their collective well-being, beneficial changes can happen [16, 17]. Community engagement strategies have a significant positive association with physical health, mental health, health behaviors, and overall wellbeing, and these strategies also enhance self-efficacy, and strengthen social support networks across diverse populations [16–18]. A national survey underscored this potential, finding that individuals with a strong sense of community were strikingly more likely to participate in health-related civic activities and engaged over 20% more in health initiatives compared to those who felt disconnected [16]. This connection is not coincidental - it demonstrates how power dynamics fundamentally shape an individual’s perception of their ability to create change.

### Theoretical Framework: The Three Faces of Power

The Three Faces of Power framework offers a detailed lens for understanding power dynamics in social and political contexts. The framework, initially proposed by Lukes as The Three Dimensional View of Power, was subsequently operationalized for application in community organizing, social justice and political advocacy, and citizen participation [19–21]. The three faces of power include visible, hidden, and invisible power dynamics [20, 21]. Visible power is defined as observable decision-making bodies, institutions, and formal political processes. This face of power is most apparent in government structures, legislative bodies, and official policy-making mechanisms, where decisions are made through transparent, recognized channels such as voting, parliamentary procedures, and public hearings [20, 21]. A city council meeting during which policies are debated and voted on is visible power in action. Hidden power involves the ability to set political agendas, determine which issues are discussed or ignored, and who is represented or excluded during the decision-making process [20, 21]. This face of power works beneath the surface, like a nonprofit organization that informs community projects, leads promotional or awareness campaigns, and advocates for certain issues. It is about who decides what problems get attention, which voices are heard, and which concerns are pushed to the margins. Invisible power shapes people’s beliefs, consciousness, and understanding of their social position through deeply embedded cultural norms and psychological conditioning [20, 21]. This is the most deeply rooted form of power, operating in the realm of personal beliefs and societal expectations. It is the power that makes people internalize limiting beliefs about themselves or accept unequal systems as the norm.

### Community Inclusion, Co-Creation, and Participatory Models

Although community engagement and involvement in public health policy, practice, and research is widely recognized as essential to achieving health equity, several challenges to successful, sustainable, and meaningful participation and power sharing amongst stakeholders remain [22–26]. Some of these challenges include limited depth of community participation, reproduction of inequities within engagement, and gaps in evaluation and sustainability. For instance, Morales-Garzón et al. found in their scoping review that communities had higher engagement in the design and implementation process of co-created initiatives compared to agenda setting and evaluation processes, with only a few studies showing community involvement across the whole decision-making scale [24]. Furthermore, delving into a commonly used population-based health tool, Ravaghi et al. found in their scoping review of Community Health Needs and Assets Assessments (CHNAAs) that there is growing interest and use of participatory approaches when conducting CHNAAs [27].

However, this approach is not fully integrated yet, as the majority of studies involve consultation-only community participation, compared to more in-depth moderate or extensive participation [27]. The studies that used participatory approaches reported a broader and deeper understanding of the concerns of marginalized and vulnerable populations typically missed in traditional health needs assessment methodologies [27]. Additionally, when using participatory approaches, voices of privilege in the community are sometimes centered during co-creation and participatory research, and marginalized voices are further excluded, maintaining power dynamics [26]. This weakens confidence, diminishes the impact of interventions, and sustains those exact injustices that public health seeks to change.

Lastly, Health Impact Assessments (HIAs), a significant tool in the Health in All Policies (HiAP) approach assesses the potential health effects of non-health policies, programs, and projects across different sectors (education, employment, etc.), contributing to the promotion of health-enhancing considerations [28]. Most HIA guidelines emphasize community engagement as a core element, a view that is generally, but not unanimously reflected in research [28]. Additionally, research shows that community participation in HIAs yield positive results and experiences including adding local knowledge that strengthens the evidence base, supporting local democracy and equitable decision-making, empowering disadvantaged groups, and supporting relations between communities, policy makers, and local stakeholders [28]. However, concerns are expressed about the lack of long term evaluation of these impacts on communities and an emphasis on pragmatic methods in community participation rather than systematic, evidence-based processes [28]. Overall, these findings suggest that while participatory models are increasingly used, they often are insufficient in meaningfully redistributing power across the visible, hidden, and invisible domains.

### Study Context and Relevance

The Equity in Public Health Initiative (EPHI) in Greenville County, South Carolina, was launched to address persistent structural inequities and health disparities disproportionately affecting Black and Hispanic communities [29]. These populations face elevated risks of chronic disease, early morbidity, and limited access to quality health care, education, and built environment resources [30]. Greenville County especially ranks among the lowest in the nation for upward mobility among children in high-poverty households, a reality shaped by systemic factors like housing quality, school access, and neighborhood-level opportunity [31]. Grounded in community voices, EPHI uses a power-focused framework to examine how civic engagement, institutional practices, and neighborhood conditions intersect to shape health outcomes - laying the groundwork for policy and systems change to advance racial and health equity. This study is one of many EPHI projects, and the results will be used by trusted community partners, including a local non-profit organization, in conjunction with the EPHI advisory board to implement actionable steps in Greenville County, South Carolina .

### Study Aim

This study aims to explore the three faces of power (visible, hidden, and invisible) through focus groups with Black and Hispanic residents and stakeholder interviews with decision-makers in Greenville County, South Carolina. The focus groups examined residents’ perceptions of power, civic engagement, government, community organizing, and trust, while the interviews explored how stakeholders understand community engagement and power dynamics. We build on research highlighting the importance of community power and PDOH to improving health inequities and the limitations of current participatory approaches by seeking to (1) understand how power is perceived, experienced, negotiated, and enacted by two communities impacted by health inequities in Greenville County, and (2) identify gaps and alignments between community and stakeholder perspectives about power and engagement. Through this research, we hope to advance community-driven approaches that move beyond consultation toward meaningful redistribution of power and structural change. This work contributes to the literature on community power by operationalizing theoretical constructs of power within real-world public health systems.

## Methods

The present analysis includes data from seven focus groups [three with Black participants (2 adult and 1 youth group) and four with Hispanic participants (3 adult and 1 youth group)] totaling 68 individuals, as well as 16 stakeholder interviews, for a combined sample of 84 participants. For the seven focus groups, interviews were conducted between March and June 2024. Participants were eligible if they: (1) identified as a Black or Hispanic; (2) lived in the specified areas in Greenville County, South Carolina (SC); (3) were either youth (ages 14–17) or adults (18 and older); and (4) were available to participate in a 90-minute focus group conducted in English or Spanish based on the participant’s preference. Stakeholder interviews were conducted between September and December 2024 with 16 stakeholders who held leadership roles within Greenville County. These included: 6 elected official stakeholders, 6 government official stakeholders (staff at city and county level government or adjacent organizations), and 4 advocacy group stakeholders (non-profit organizations with advocacy priorities).

Purposive sampling was used to ensure a balanced representation across the two racial and ethnic groups included in this study, with the intention of capturing varied perspectives. The focus group participants were recruited using multiple community engaged strategies including through existing networks, word of mouth and active partnerships with the Racial Equity and Economic Mobility (REEM) Commission and the Hispanic Alliance. The 90-minute focus groups were conducted in person in community spaces. Participants received a $50 Walmart gift card for participation and completed a short demographic survey. For stakeholder interviews, the EPHI advisory board and research team curated a list of potential stakeholders. The research team invited the potential stakeholders to participate in a 30-45-minute interview on Zoom. The participants were offered a $50 Walmart or Amazon gift card, which most donated back to the study.

This study was approved by the Furman University Institutional Review Board. Most focus groups were racially homogeneous, including race-matched trained moderators. A semi-structured focus group guide was developed based on a review of the literature and the study’s research aims to explore participants’ (focus groups) perceptions of their experiences related to power, civic engagement and trust, visible power, hidden power and invisible power, community organizing and health. The subset of questions on the guide were as follows: *Power*,* Civic Engagement and Trust -* “How do you use community power? If you don’t currently use it, how would you use it?”. *Visible Power -* “Who holds decision-making power in your community? What are examples of decisions that they made that affected the community?”. *Hidden Power -* “How can non-profit organizations, community organizations, or leaders build trust?”. *Invisible Power -* “What shapes worldviews in Greenville County?”. *Community Organizing -* “How do you think a leader could keep the interest of their base over a long period of time?”. *Health -* “Thinking about everything we discussed - power, civic engagement, government, community organizing, and trust; how does it impact the health of people in your community?”.

The guide for stakeholders highlighted the themes of civic engagement, client engagement, economic mobility, built environment, government transparency and community power looking deeply into the barriers and strategies that stakeholders addressed and recommended. The subset of questions on the guide were as follows: *Civic Engagement -* “What strategies do you employ to address barriers to civic engagement from constituents from marginalized groups?”. *Client engagement -* “How would you describe your role in engaging your clients/constituents?”. *Economic Mobility -* “What barriers to economic mobility have you observed in your role? How does your organization address economic mobility? What strategies would you add?” *Built Environment -* “What barriers to an improved built environment have you observed in your role? How does your organization foster the built environment? What strategies would you add?” *Government Transparency -* “What barriers to transparency in local government have you observed in your role? How does your organization foster transparency in local government? What strategies would you add?” *Community Power -* “How do you see your organization engaging with community power building?”.

To prepare for analysis, the sessions were audio-recorded, transcribed, and de-identified. The research team developed a codebook based on the categories outlined in the semi-structured focus group guide and stakeholder interview guide. Additional codes were added inductively as new concepts emerged from the data. NVivo was utilized for data management. For focus groups, one team member coded the data in NVivo while at least one other team member was present for the coding of each of the seven transcripts. This collaborative approach was chosen to facilitate discussions and ensure complete consensus on the most accurate categorization of the data. This collaborative coding process enhanced analytical rigor and allowed for immediate reflection on group dynamics and emergent insights. For the stakeholder interviews, the data was double coded, and once 80% agreement was reached, using the NVivo Coding Comparison Query, one coder completed the rest of the transcripts. Since the interviews were one-on-one conversations, the research team employed double coding to ensure consistency in interpretation across coders, as the absence of group interaction made it more important to independently validate how key themes were identified and interpreted.

Once all transcripts were coded, the team generated coding reports in NVivo. These reports compiled all text segments associated with each individual code. To enable comparative analysis, the coding reports were disaggregated by focus group type: Black focus groups, Hispanic focus groups, elected official stakeholder interviews, government official stakeholder interviews, and advocacy stakeholder interviews. Using a thematic analysis approach guided by Clarke and Braun framework, the research team reviewed the coding reports through a series of iterative meetings [32]. Key themes were identified and refined by examining patterns both within and across participant groups, with attention to areas of convergence and divergence in participants’ experiences. This approach facilitated an exploration of how power was shaped by varying positions within the political system. Final themes were confirmed through team discussions to ensure consensus on the thematic structure [32].

The data collection and analysis team was composed of individuals who identified as Black, Hispanic, Asian, and White. The team brought diverse lived experiences and professional expertise to the research process, which informed the interpretation of participant narratives. One member of the research team is an elected official, and others have worked across various levels of community engagement, advocacy, and institutional leadership. These perspectives provided insight into the dynamics of power and policymaking explored in the study.

## Results

### Participants

Table 1 has the demographic information for the 68 focus group participants. The majority of the sample identified as Latino/Hispanic, followed by Black/African American. The participants were predominantly female and between the ages of 36–45 years. The most recurring highest level of education among participants was high school graduate or equivalent degree (GED), and the most recurring income level was a household income of $20,000 - $29,999 USD per year. Similarly, the majority of participants worked either full-time or part-time. More participants were born inside of the US compared to outside of the US, spoke Spanish at home compared to English, and had not voted in the past 12months. The study also included 16 stakeholder interview participants spanning 6 elected official stakeholders, 6 government official stakeholders (staff at city and county level government or adjacent organizations), and 4 advocacy group stakeholders (including philanthropy and faith communities).

**Table 1:** Equity in public health initiative (EPHI) demographic table. Demographic information for focus group participants (*n* = 68)

Categories	Black % (*n*)	Hispanic % (*n*)	Total % (*n*)
*Gender*			
Male	2.94% (*n*=2)	16.18% (*n*=11)	19.12% (*n*=13)
Female	38.24% (*n*=26)	41.18% (*n*=28)	79.41% (*n*=54)
Prefer Not to Say	0% (*n*=0)	0% (*n*=0)	0% (*n*=0)
No Response	0% (*n*=0)	1.47% (*n*=1)	1.47% (*n*=1)
*Age*			
14-17 years old	1.47% (*n*=1)	8.82% (*n*=6)	10.29% (*n*=7)
18-24 years old	7.35% (*n*=5)	5.88% (*n*=4)	13.24% (*n*=9)
25-35 years old	4.41% (*n*=3)	5.88% (*n*=4)	10.29% (*n*=7)
36-45 years old	5.88% (*n*=4)	22.06% (*n*=15)	27.94% (*n*=19)
46-55 years old	8.82% (*n*=6)	16.18% (*n*=11)	25.00% (*n*=17)
56-65 years old	5.88% (*n*=4)	0% (*n*=0)	5.88% (*n*=4)
65+ years old	7.35% (*n*=5)	0% (*n*=0)	7.35% (*n*=5)
Prefer Not to Say	0% (*n*=0)	0% (*n*=0)	0% (*n*=0)
*Education*			
Less than High School	0% (*n*=0)	5.88% (*n*=4)	5.88% (*n*=4)
Some High School	1.47% (*n*=1)	11.77% (*n*=8)	13.24% (*n*=9)
High School Graduated or Equivalent Degree GED)	7.35% (*n*=5)	22.06% (*n*=15)	29.41% (*n*=20)
Some University or Technical School, but no degree	11.77% (*n*=8)	14.71% (*n*=10)	26.47% (*n*=18)
Two-Year University Degree	4.41% (*n*=3)	0% (*n*=0)	4.41% (*n*=3)
Four-Year University Degree	5.88% (*n*=4)	2.94% (*n*=2)	8.82% (*n*=6)
Graduate School (Doctorate, Masters, etc.)	10.29% (*n*=7)	0% (*n*=0)	10.29% (*n*=7)
Prefer Not to Say	0% (*n*=0)	1.47% (*n*=1)	1.47% (*n*=1)
Employment*			
Working Full-Time	17.65% (*n*=12)	14.71% (*n*=10)	32.35% (*n*=22)
Working Part-Time	11.77% (*n*=8)	19.12% (*n*=13)	30.88% (*n*=21)
Self-Employed	7.35% (*n*=5)	7.35% (*n*=5)	14.71% (*n*=10)
Stay-at-Home Parent	1.47% (*n*=1)	16.18% (*n*=11)	17.65% (*n*=12)
Student	5.88% (*n*=4)	4.41% (*n*=3)	10.29% (*n*=7)
Not Employed, Looking for Work	1.47% (*n*=1)	0% (*n*=0)	1.47% (*n*=1)
Not Employed, Not Looking for Work	0% (*n*=0)	0% (*n*=0)	0% (*n*=0)
Retired	8.82% (*n*=6)	0% (*n*=0)	8.82% (*n*=6)
Not Working Due to Disability	0% (*n*=0)	0% (*n*=0)	0% (*n*=0)
Prefer Not to Say	0% (*n*=0)	0% (*n*=0)	0% (*n*=0)
*Household Income*			
Less than $10,000	4.41% (*n*=3)	17.65% (*n*=12)	22.06% (*n*=15)
$10,000 - $19,999	1.47% (*n*=1)	1.47% (*n*=1)	2.94% (*n*=2)
$20,000 - $29,999	7.35% (*n*=5)	7.35% (*n*=5)	24.71% (*n*=10)
$30,000 - $39,999	7.35% (*n*=5)	2.94% (*n*=2)	10.29% (*n*=7)
$40,000 - $49,999	1.47% (*n*=1)	10.29% (*n*=7)	11.77% (*n*=8)
$50,000 - $59,999	4.41% (*n*=3)	2.94% (*n*=2)	7.35% (*n*=5)
$60,000 - $69,999	2.94% (*n*=2)	1.47% (*n*=1)	4.41% (*n*=3)
$70,000 - $79,999	0% (*n*=0)	0% (*n*=0)	0% (*n*=0)
$80,000 - $89,999	4.41% (*n*=3)	0% (*n*=0)	4.41% (*n*=3)
$90,000 - $99,999	0% (*n*=0)	1.47% (*n*=1)	1.47% (*n*=1)
More than $100,000	4.41% (*n*=3)	2.94% (*n*=2)	7.35% (*n*=5)
I don’t know	1.47% (*n*=1)	10.29% (*n*=7)	11.77% (*n*=8)
Prefer Not to Say	1.47% (*n*=1)	0% (*n*=0)	1.47% (*n*=1)
*Race/Ethnicity*			
Latino/Hispanic	0% (*n*=0)	58.82% (*n*=40)	58.82% (*n*=40)
Black/African American	39.71% (*n*=27)	0% (*n*=0)	39.71% (*n*=27)
Asian American/Asian from the Pacific Islands	0% (*n*=0)	0% (*n*=0)	0% (*n*=0)
Native American/Indigenous	0% (*n*=0)	0% (*n*=0)	0% (*n*=0)
White/Caucasian	0% (*n*=0)	0% (*n*=0)	0% (*n*=0)
Unknown	0% (*n*=0)	0% (*n*=0)	0% (*n*=0)
Other	0% (*n*=0)	0% (*n*=0)	0% (*n*=0)
Prefer Not to Say	1.47% (*n*=1)	0% (*n*=0)	1.47% (*n*=1)
*Geographic Location – Were you born in the United States?*			
Born in the United States	41.18% (*n*=28)	14.71% (*n*=10)	55.88% (*n*=38)
Born outside the United States	0% (*n*=0)	33.82% (*n*=23)	33.82% (*n*=23)
Prefer Not to Say	0% (*n*=0)	10.29% (*n*=7)	10.29% (*n*=7)
*Language – What language do you prefer to speak at home?*			
English	39.71% (*n*=27)	5.88% (*n*=4)	45.59% (*n*=31)
Spanish	0% (*n*=0)	51.47% (*n*=35)	51.47% (*n*=35)
No Response	1.47% (*n*=1)	1.47% (*n*=1)	2.94% (*n*=2)
Voting – Have you voted in the past 12 months?			
Yes	25.00% (*n*=17)	2.94% (*n*=2)	27.94% (*n*=19)
No	14.71% (*n*=10)	45.59% (*n*=31)	60.29% (*n*=41)
Prefer Not to Say	1.47% (*n*=1)	10.29% (*n*=7)	11.77% (*n*=8)
Voting – What elections have you ever voted in?*			
Primary Election	23.53% (*n*=16)	2.94% (*n*=2)	26.47% (*n*=18)
General Election	20.59% (*n*=14)	2.94% (*n*=2)	23.53% (*n*=16)
Prefer Not to Say	4.41% (*n*=3)	45.59% (*n*=31)	50.00% (*n*=34)
Not Applicable (never voted)	11.77% (*n*=8)	8.82% (*n*=6)	20.59% (*n*=14)

*Participants could select multiple responses; therefore, percentages may total more than 100%.

### Themes

#### Perceptions of Power

Focus group (FG) participants discussed power perceptions as the ways individuals and communities view their ability to influence change. Some see power as residing within their own community (internal locus of control), while others perceive it as held by external authorities like government or corporations (external locus of control). These perceptions shape how people engage with power, with those who view power as internal actively working for change, while others may feel reliant on external forces like governmental support or leadership. One participant’s perception of power was that, “I think the power one has is being able to do what is within one’s possibilities, without deceiving anyone else. That’s it” (FG1, Hispanic Adults).

Additionally, FG participants identified community power as the collective strength that enables groups to influence change, either through direct action or by shaping public discourse and policy. As one participant shared, “I feel like community power can potentially be one of the strongest powers because now you don’t have one individual trying to call the shots. You have people on the same page, one in the same thing, to make a change” (FG7, Black Youth). Another participant also echoed this perspective stating, “I believe that the community power is used to fulfill our needs. When we need to be heard, we come together because several people are better than just one. We come together to achieve a purpose and be heard by a person who has greater power than us” (FG2, Hispanic Adults). Participants also shared that community organizations can serve as catalysts for uniting people and channeling their concerns into action. They mobilize resources, build relationships, and amplify community voices to advocate for needs in the broader socio-political landscape.

Similarly, stakeholder interview (SI) participants described community power as the ability of individuals and communities to influence decisions, shape services, and have their voices respected in public processes. At its core, community power is about having a voice (i.e., being heard, recognized, and included). Some emphasized this power as collective and grassroots, like one participant who shared, “Community power to me looks like the every average day people coming together, working together and having a voice to make change in their community” (Interview 4, Advocacy Stakeholder). Others noted that power is unevenly distributed, often residing with the upper middle class or those who are most civically engaged. Still, several interviewees highlighted that community power is a latent force, like “political potential energy” (Interview 6, Elected Official) that exists whether people use it or not, and its impact depends on how it is activated and shared across all levels of a community.

Participants described community power building as a dynamic and ongoing process of equipping individuals and groups with the knowledge, resources, relationships, and confidence to influence change. Participants expressed that the community power building process requires intentional investments such as information sharing on relevant topics, leadership training, and community infrastructure including neighborhood associations, child care, and compensation for time and labor. At its best, power building fosters a sense of collective agency, where residents recognize their ability to lead, advocate, and take sustained action. The barriers to community power were identified as limited access to information, systemic inequities, lack of civic education, resource constraints, disenfranchisement, and policy limitations. One participant specified how these barriers create exhaustion among community members and lead to disinterest in community power building by sharing, “People are made to stay tired… if you’re on the bus four hours a day just to work and survive, how can you show up to advocate about street lights?” (Interview 11, Advocacy Stakeholder).

### Visible Power

#### Government, Policy, & Politics

Many FG participants described a disconnect between government officials and their communities, especially among marginalized groups. A major concern was that officials rarely engage youth in meaningful ways, missing opportunities to build trust with younger generations. Social media was identified as an essential tool for creating more relatable leaders as one participant said, “Seeing younger politicians interact through social media … I feel like that gains more trust because it is relatable” (FG4, Hispanic Youth). Participants also highlighted disparities in resource allocation, describing frustration over seeing wealthier neighborhoods prioritized in receiving services while lower-income areas were neglected. One participant stated, “they cleaned the houses where the nice people with money lived… For mobile homes, we had to do it ourselves” (FG2, Hispanic Adults). These experiences reinforce perceptions of inequity and contribute to feelings of exclusion from local decision making. Another issue that surfaced throughout the discussion was the varying levels of trust in elected officials and the police. Some FG participants felt protected, like an individual who shared, “I felt confident calling 911 for the police to arrive” (FG1, Hispanic Adults), while others described systemic failures that unfairly impacted minorities noting, “Minorities suffer the most because of the justice system’s failures” (FG2, Hispanic Adults).

#### Engagement Beyond City Hall

Several SI participants echoed the disconnect between government officials and their communities, as described by FG participants. Stakeholders noted that the local government should expand its engagement efforts beyond formal council meetings. Suggestions included holding meetings in community spaces like parks or organizing door-to-door visits to better connect with residents. Stakeholders advocated for increased visibility of council members in everyday community settings, arguing that such engagement would help bridge the gap between government and citizens, fostering stronger relationships and ensuring that the voices of residents were heard. One stakeholder stated, “Yeah, I think engagement outside the walls of city hall and the county chambers would be a part of that. Whether it’s holding workshops at community spaces. As I mentioned before, we’ve got wonderful parks and recreation facilities where meetings could be convened in the neighborhood at different hours of the day so that many people could participate. And I think that some of our resources could be used to do a better job of being out in the community on foot, engaging neighbors door to door. People want to know relationally that they are a part of that process. And when we build those relationships, that’s when people have more confidence in the process.” (Interview 7, Advocacy Stakeholder).

#### Civic Responsibility

FG participants discussed civic responsibility as an individual action and collective participation, with voting viewed as an important, though sometimes insufficient, tool for influence. Some described voting as a starting point that should lead to deeper community involvement and engagement with government officials. A shift from formal political participation to visible community action was highlighted, as participants explained that showing up at local meetings helped empower their representatives. One participant said, “In order for them to actually hear what [our state representative] has to say and consider it, we as the community have to empower him by showing up to zone hearings, meetings, so they can see that we are interested” (FG5, Black Adults).

#### Civic Engagement

Most SI participants described civic engagement in Greenville as a fragmented and inequitable system shaped by economic stratification, transportation barriers, and generational divides. Lower-income residents were often excluded not because of disinterest, but because basic survival needs left little time or resources for political participation. As one government official explained, childcare had to be offered just to help working parents attend meetings (Interview 14, Government Official). Transportation challenges compounded this exclusion because without reliable public transit, many residents simply could not reach civic events or access local government services. “Engaging civically, there’s not a bus stop on the side of the bridge… Scheduling are barriers, time of day when people have available time to participate doesn’t always coincide with the availability of public transportation” (Interview 7, Advocacy Stakeholder). Participants also described Greenville’s institutional communication gaps, with government entities and developers struggling to consistently share information. Open house formats were introduced as a response to outdated neighborhood meetings that often left residents unheard, and this has had some positive impact. As one participant mentioned, “By going to this new format… there’s a lot more honest and sincere one-on-one communication” (Interview 15, Government Official).

SI participants noted that schools no longer emphasize civics, leading to confusion about government processes and weakening traditional civic structures like neighborhood associations and churches. Generational patterns further complicated engagement with older residents shaped by the civil rights movement maintaining strong civic participation, while younger people showed growing but underdeveloped interest in political involvement. One participant noted, “There is a growing movement among the young people… The older population is still very much engaged because they came through a different time period” (Interview 1, Elected Official). Throughout, communication itself emerged as a strategy for civic engagement, but mistrust remained high, with many viewing local political communication as futile. One participant shared “I think the biggest barrier is the belief that somehow involvement or communication with elected officials, especially at the local level, isn’t going to be of benefit… I probably was what I call a political agnostic. I believed it existed, but I didn’t know how it existed or what it did or how it even benefited me in any way” (Interview 6, Elected Official).

#### Built Environment

Participants across focus groups and stakeholder interviews highlighted how infrastructure development and urban planning serve as visible demonstrations of power, directly shaping accessibility and quality of life in their communities. Many noted positive investments such as parks and sidewalks, yet expressed frustration that these improvements disproportionately benefit wealthier neighborhoods, leaving marginalized areas overlooked. As one participant observed, “It’s great to see the city investing in parks and sidewalks, but why do they only seem to appear in wealthier areas?” (FG3, Black Adults). Another participant echoed this concern, stating, “They build up downtown for the tourists, but where we live, nothing changes” (FG4, Hispanic Youth). Others described physical barriers, like cement dividers, that hinder local access. A stakeholder reflected similar concerns, noting, “Development always starts where the money is. Poor neighborhoods are left waiting for infrastructure that never comes” (Interview 7, Advocacy Stakeholder).

Discussions of growth and expansion often centered on gentrification and displacement, with participants noting that development projects prioritize businesses and higher-income residents over environmental preservation and community needs. Stakeholders stressed the need for reforming zoning policies, expanding mixed-use developments, and incentivizing quality affordable housing to ensure that infrastructure growth benefits all residents, not just the privileged few. Poor coordination between government entities was also identified as a challenge, slowing infrastructure progress and contributing to fragmented growth that fails to address community priorities.

#### Government Transparency

FG participants and stakeholders consistently described limited transparency in local government, citing a closed culture that makes accessing public information difficult and navigating government processes confusing. Some noted positive changes, like increased use of online videos, but overall found the system inaccessible. As one government stakeholder explained, “It depends on the government partner, but there can be a lot of gatekeeping. There can be a lot of, it’s hard to find stuff on the website, it’s hard to find minutes, it’s hard to find agendas, that kind of thing” (Interview 14, Government Official). Beyond structural barriers, participants perceived an intentional unwillingness to share power among long-standing officials who prefer to keep decision-making centralized. One elected official described this dynamic as “deliberate chaos,” explaining, “when chaos exists, then it allows for the consolidation of power at the top… Many people find out decisions that have been made after the fact, even though technically you should have been part of that discussion” (Interview 1, Elected official). A lack of centralized, easily accessible political information was also highlighted as a key factor preventing civic participation and reinforcing distrust in local government.

### Hidden Power

#### Bridges

FG participants described organizations as essential “bridges” connecting communities to government, helping translate local needs into priorities visible to decision-makers. Churches, schools, social media, and minority-serving organizations were seen as key players in this hidden power structure. Churches, in particular, were recognized for their ability to organize communities and facilitate communication between residents and local officials, with one participant noting, “We’re here and the government is over there. They act like a bridge. That’s how we reach them” (FG1, Hispanic Adults). Another participant explained, “I imagine the government goes to the churches and asks what the communities need, or the churches join forces and have some influence somehow” (FG3, Hispanic Adults). Schools were viewed as critical spaces for empowering youth to lead change, while social media platforms like Facebook and WhatsApp provided accessible tools for sharing information and mobilizing communities. Community organizations were highlighted as essential advocates for marginalized populations, offering both resources and relationships needed to strengthen community power and influence.

#### Community Organizing

FG participants expressed strong support for community organizing, seeing it as a collective and necessary process for driving change in their communities. Many emphasized that personal involvement is essential, with one participant explaining, “I’m here because it matters to me… I’d like to learn and maybe even train to do something different from what we’ve been doing for so many years and be able to progress as a community” (FG3, Hispanic Adults). Organizing was described as a collaborative effort where community members come together to identify priorities and set shared goals. A participant shared that “It’s a community getting together on one accord, outlining things, decisions, goals, and objectives that the community wants to make as a group” (FG6, Black Adults). Trust-building was recognized as critical to sustaining participation, as leaders need to fulfill promises and demonstrate transparency to maintain engagement. Participants also emphasized the importance of educating community members about the issues they face, noting that understanding the relevance of a cause increases motivation and long-term involvement. As one participant stated, “If it’s a cause that not only do I relate to, but I’ve explained how it has been or what the problem with it is, I feel like that would convince me to stay longer” (FG4, Hispanic Youth).

### Invisible Power

#### Race and Ethnicity – Unity and Division

Many FG participants emphasized the dual role of race and ethnicity in shaping both community cohesion and intergroup tension. While some highlighted strong internal unity, particularly within the Hispanic community, others noted persistent divides, both between and within racial groups. A participant observed how Hispanic residents mobilized economically despite external mockery, saying, “That community had one standard. That was to build. While others sat back watching, they stood on their principles of group economics and they marked their territory” (FG6, Black Adults). In contrast, others reflected on enduring racial privilege and inequity. One participant remarked, “Even if you are a poor White person, you still have that privilege… no Caucasian person is going to fight for what’s best for the Black community like a Black person would” (FG7, Black Youth). These insights underscore the complexity of racial dynamics, where solidarity often emerges in response to systemic division.

#### Immigration and Language – Alienation and Trust Issue

Participants, especially Hispanic FG voices, shared feelings of alienation rooted in immigration status, language barriers, and systemic exclusion. A participant noted how the language of immigration law dehumanizes: “We’re not seen as people, we’re seen as aliens” (FG4, Hispanic Youth). Beyond legal labels, the experience of cultural division was also deeply felt, as one participant described the racialized geography of Greenville as “The ‘us’ and ‘them’” (FG6, Black Adults). These narratives reveal the psychological and social toll of being perceived as outsiders, often accompanied by mistrust toward institutions and civic processes.

#### Socioeconomic Status (SES) – Power and Division

Discussions around socioeconomic status emphasized how deeply class impacts power, choice, and visibility in the community. FG Participants consistently pointed to the structural nature of economic inequality. One participant reflected, “The haves and the have-nots… paying other people to speak up so they can step into the background,” illustrating how wealth influences both political expression and social positioning (FG6, Black Adults). Another highlighted the inevitability of economic stratification saying, “It’s set up where 100% of the community cannot be wealthy. You have to have those tiers” (FG5, Black Adults). These perspectives convey a sense of systemic entrenchment, where SES is more than a financial category—it defines one’s access to voice, resources, and agency.

#### Economic Mobility

SI participants linked economic mobility to a web of systemic barriers, particularly inadequate transportation, substandard education, and unaffordable housing. Transportation was framed as a foundational issue. One participant mentioned, “Economic mobility goes hand in hand with transportation. It’s all interconnected” (Interview 13, Elected official). Similarly, education was described as structurally unequal, shaped by socioeconomic and policy factors like school vouchers that redirect resources away from low-income communities. A participant noted, “You’re relying upon the school buses… You’re only going to get $6,000, that tuition is $18,000, so that’s going to disqualify you anyway” (interview 1, Elected Official). They also emphasized that poverty is not just a lack of resources but a condition that generates additional burdens. As one government stakeholder explained, “If you study people who, one, are historically marginalized and two, come from low-wealth families, it’s these consistent issues that pop up that make it so they can never get ahead. It’s very expensive to be poor” (Interview 3, Government Official). This sentiment reflects how ongoing financial instability, systemic disadvantages, and unexpected costs can prevent families from building long-term stability. Housing was also seen as a critical but increasingly inaccessible pathway to stability, hindered by inflation, exclusionary approval processes, and low investment incentives for developers. Together, these interlocking barriers create a feedback loop that keeps those already disadvantaged from accessing the very tools, mobility, education, and housing that could support long-term economic advancement.

## Discussion

Using the Three Faces of Power framework, we explored Black and Hispanic residents and community stakeholders’ perceptions of power, civic engagement, government, community organizing, trust, and community engagement in Greenville County, South Carolina. Our qualitative analyses of focus groups with residents and interviews with stakeholders showed that visible power was marked by government distrust and inequitable access to political engagement and public resources, as experienced by marginalized communities. Participants highlighted the significance of hidden power in their communities, embodied through faith communities, schools, social media platforms, and nonprofit and other community organizations. These institutions were described as a “bridge” between residents and local government, crucial for the remediation of challenges described under visible power. Participants also described key invisible drivers of power including socioeconomic status (SES), race and ethnicity, and immigration and language.

Overall, findings from community members and stakeholders reflect how political determinants of health (PDOH) structure who holds decision-making power and whose priorities are translated into policy. Participants’ descriptions of inequitable resource allocation, limited government transparency, and barriers to civic participation illustrate how policy decisions and institutional practices actively shape the conditions for health and civic engagement in Greenville County, SC. Our findings underscore a persistent imbalance in community power, particularly in the limited opportunities for co-governance and shared decision-making. Although one important area of alignment between stakeholders and community members was a shared recognition of structural barriers (e.g., transportation, childcare, and communication gaps), these challenges are not currently addressed through mechanisms that meaningfully redistribute power or deepen community involvement in decision-making processes.

By contrast, a notable area of misalignment was the difference in interpretation of power, civic engagement, and structural barriers. While both groups described similar issues, community members emphasized lived experiences of exclusion and how structural inequities constrain their ability to participate meaningfully in decision-making processes. In contrast, stakeholders more frequently framed these same barriers as procedural, logistical, or programmatic challenges, emphasizing solutions such as expanding outreach and modifying engagement formats. Additionally, stakeholders demonstrated a strong understanding of structural issues, particularly those related to socioeconomic status, but less frequently discussed challenges based on race, ethnicity, and immigration status, as voiced by community members.

### Visible Power

Many of our participants indicated that Greenville’s infrastructure development, distribution of public resources, civic engagement, and access to decision-making are shaped by visible yet uneven exercises of power, and that achieving equity across race, ethnicity, and socioeconomic status requires deliberate action from both government and community members. In our discussions, participants described the need for more equitable allocation of resources and development projects among all communities, giving as much priority to lower income neighborhoods as compared to wealthier ones. SI participants specifically acknowledged the role of restrictive and outdated zoning practices as a barrier to balanced development. In several instances, FG and SI participants identified challenges within the realm of visible power and even offered solutions, yet implementation remains a challenge. These findings show how governmental actions such as compliance with policies that create inequitable distribution of resources for marginalized communities can serve as a political determinant which inhibits the health of a population.

This aligns with a perspective offered by House-Niamke and Eckerd (2020) on how public administration fosters racial disparities through its policy-making, and an alternative pathway to address this issue. They shed light on the oversimplification and dichotomizing of race itself as a problem, highlighting that communities should not be understood as either Black or White based on the majority racial demography of residents. Instead, stakeholders should focus on the complex intersectionality of multiple components of the residents’ identities and not just aggregate individuals based on the incomplete measurement of skin pigmentation, without an understanding of backgrounds and life experiences [33]. This approach can create tailored and community-engaged solutions for communities, which can be implemented through minor system disruptions, contrary to the common misconception of an unchanging political system [33].

Additionally, participants highlighted the value of building stronger connections and pathways for engagement between government officials and community members as a way of rebuilding trust. To foster this engagement and promote change, participants also emphasized mutual respect and responsibility, wherein marginalized voices can champion the changes they hope to effect, and elected officials can act upon those changes transparently while being held to standards of accountability. Plus, genuine effort is applied to address the structural barriers that even prevent engagement such as transportation. FG participants also talked about the importance of voting as a powerful way to influence governance and hold officials accountable, though they pointed out barriers to voter participation such as misinformation and a lack of outreach. This was evident in the higher percentage of FG participants who had not voted within the past 12 months. Indeed, it seems that building equitable environments requires intentionality, and shifting these visible power dynamics could influence not only the quality of our public spaces but also our collective participation in civic life.

These findings align with research which emphasizes the need for more equitable applications of visible power and the promotion of community engagement. Mittal and Bansal discusses that when stakeholders allocate resources and provide financial incentives to cover childcare, transportation, and other services, it can bridge the socioeconomic divide in civic and community engagement [34]. Additionally, they acknowledge that stakeholders must apply democratic strategies to ensure a diverse demography of participants, promote transparency throughout the engagement process, and empower communities through joint decision-making rather than limiting engagement to surface level [34].

One field model is The Neighborhood Health Initiative (NHI) which demonstrates how challenging power structures through initiatives that create spaces for collective action and decision-making can create meaningful change [35]. These initiatives transform the underlying power dynamics that shape health outcomes and community well-being. NHI offers a powerful example of challenging power structures by intentionally bringing together a diverse group of community members (i.e., immigrants, formerly incarcerated individuals, and people from various racial and ethnic backgrounds). They created a model of inclusive decision-making, which confronts the power dynamics that traditionally silence marginalized communities.

### Hidden Power

In our study, participants described faith communities, schools, social media platforms, and minority-serving organizations as essential “bridges” connecting residents to local government, reflecting research on the political influence of faith-based networks and their role in community power structures. Studies have shown that evangelical Christians have exerted significant political influence over the past three decades, primarily in conservative causes [36–38], but faith-based engagement also spans the political spectrum, particularly in poorer areas where congregations support grassroots organizing and civil society development [36, 39]. This dual capacity was reflected in our focus groups, where churches were seen as trusted spaces for organizing and amplifying community needs.

Participants also observed that government partnerships with faith-based organizations (FBOs) could enhance service delivery and community development, echoing policy trends toward such collaborations [40]. Within Christens’ multidimensional framework, these institutions embody hidden power by operating behind the scenes to shape decision-making and community priorities [41]. Schools were highlighted as critical spaces for empowering youth to lead change, while platforms like Facebook and WhatsApp facilitated rapid communication and mobilization. Across these discussions, community organizing emerged as a collective process rooted in trust, transparency, and shared goals, with the emphasis that understanding the relevance of a cause sustains engagement.

### Invisible Power

Participants described socioeconomic status (SES) as a deeply entrenched driver of power, visibility, and opportunity in the community, echoing research on the invisible power dynamics of social stratification [42]. Focus group narratives reflected Chetty et al. finding that economic connectedness—relationships between low- and high-SES individuals—predicts upward mobility, as participants emphasized how wealth often determines access to influence, networks, and decision-making spaces [31]. With access to decision making, higher SES individuals can more easily address concerns that protect their interests. Participants’ lived experiences align with Wang et al.’s observation that higher-SES individuals interact less with others overall but devote more time to selective social ties, often reinforcing exclusion [43]. Participants also described the compounding nature of poverty which restricts economic mobility. Together, these perspectives illustrate how structural and interpersonal mechanisms combine to create a feedback loop that keeps disadvantaged residents from accessing the very resources that could enable long-term stability. Participants also discussed race, ethnicity, and immigration status as worldviews that promote either socio-political cohesion or tension and exclusion.

### Strengths and Limitations

Strengths of our study include the use of standardized focus groups and interviews guides, as well as racially homogenous focus groups, including race-matched trained moderators. This intentional design aimed to ensure that focus group participants felt a sense of trust and were comfortable in expressing their views and opinions. The option to participate in a focus group conducted in English or Spanish based on the participants’ choosing was also a strength. Moreover, analysis of the transcripts using established qualitative methods, such as ensuring consensus among multiple coders for the focus group transcripts and achieving 80% intercoder agreement for the stakeholder interview transcripts, were study strengths that increased academic rigor. Notably, the stakeholder interviewers had varying levels of community engagement, advocacy, and institutional leadership, including one elected official.

Limitations were present as well. The Hispanic focus groups had more participants than the Black focus groups, and there was a vast gender imbalance among FG participants which signifies the potential of missing or incomplete perspectives from the study. However, we conducted purposive sampling in conjunction with the EPHI advisory board and community partner organizations to ensure diverse representation and varying perspectives. Additionally, our study focused only on Greenville Country, South Carolina, but future studies can include an expansion to other counties within South Carolina or other states.

## Conclusion

This study uses focus groups with Black and Hispanic residents and stakeholder interviews with decision-makers in Greenville County, South Carolina to explore the three faces of power. EPHI underscores the urgent need for inclusive governance, investment in underserved areas, and community-anchored solutions to health and economic inequities.

One recommendation identified by stakeholder interview participants is to promote civic participation by offering access to transportation, childcare, and food, and addressing other barriers such as language access that hinder the engagement of marginalized community members. Another recommended solution is to improve access to civic information and education by using multiple inclusive communication channels to reach diverse audiences including door-to-door mailers, social media, transparent and user-friendly government websites, community workshops, and visually accessible documents (e.g., infographics, pie charts). Thirdly, SI participants highlighted the importance of collaborative partnerships through strategies such as hosting inclusive listening sessions, promoting collaborative planning and decision-making processes, and identifying and supporting local leaders and non-profits to amplify their community organizing efforts. A final recommendation from SI participants is promoting affordable housing by updating zoning regulations, incentivizing developers to build more inclusively, and providing housing subsidies and other rental initiatives. While these strategies represent important steps toward improving engagement and access, they alone may be insufficient to address the underlying power imbalances shaping participation and decision-making. Intentional efforts are needed to redistribute decision-making authority and embed communities in agenda-setting and governance processes, so that these approaches do not risk reinforcing existing dynamics in. Future efforts should therefore move beyond improving participation toward establishing structures for shared governance and community-led decision-making.

Our study highlights the importance of addressing economic inequalities, promoting inclusive governance and understanding the dynamics of the three faces of power as pathways to improving health and combating disparities. Through these pathways, we can aim to reduce stress and improve the physical and mental health outcomes of marginalized communities. Future studies can focus on understanding the three faces of power among multiple racial, ethnic, and other marginalized populations across South Carolina or throughout the US as a pathway to health. Future studies can also assess pilot initiative to collaborative partnerships between governments and communities based on some participant recommendations described above.

## References

[1] Farhang, L., & Morales, X. (2022). Building community power to achieve health and racial equity: Principles to guide transformative partnerships with local communities. *NAM Perspectives*. 10.31478/202206d PMC9499374 36177209

[2] Iton, A., Ross, R. K., & Tamber, P. S. (2022). Building community power to dismantle policy-based structural inequity in population health. *Health Affairs*, *41*(12), 1763–1771. 10.1377/hlthaff.2022.00540 36469831

[3] Pastor, M., Ito, J., & Wander, M. (2020). *Leading locally: A community power-building approach to structural change*. USC Dornsife Equity Research Institute. https://static1.squarespace.com/static/5ee2c6c3c085f746bd33f80e/t/5f98a9a4cd172a172549dcce/1603840428427/Leading_Locally_FULL_Report_web.pdf

[4] Pastor, M., Ito, J., Wander, M., Thomas, A. K., Moreno, C., Gonzalez, D., Yudelevitch, E., Noden, K., & Sinclair, C. (2020). *A primer on community power, place, and structural change*. USC Dornsife Equity Research Institute. https://static1.squarespace.com/static/5ee2c6c3c085f746bd33f80e/t/5f8f3a4fd196f3101aba9912/1603222096604/Primer_on_Structural_Change_web.pdf

[5] Vaidya, A., Poo, A., & Brown, L. (2022). Why community power is fundamental to advancing racial and health equity. *NAM Perspectives*. 10.31478/202206b PMC9499376 36177206

[6] Garcia, A. A., Jacobs, E. A., Mundhenk, R., Rodriguez, L., Pulido, C. L., Hall, T., Allport-Altillo, B., & Tierney, W. (2022). The Neighborhood Health Initiative: An academic–clinic–community partnership to address the social determinants of health. *Progress in Community Health Partnerships: Research Education and Action*, *16*(3), 331–338. 10.1353/cpr.2022.0050 36120876

[7] National Academies of Sciences, Engineering, and Medicine; Health and Medicine Division; Board on Population Health and Public Health Practice; Committee on Community-Based Solutions to Promote Health Equity in the United States, & Health, M. (2017). The root causes of health inequity. In A. B. Baciu, Y. Negussie, A. Geller, & J. N. Weinstein (Eds.), Communities in action: Pathways to health equity. National Academies, 99-184. https://www.ncbi.nlm.nih.gov/books/NBK425848/28418632

[8] Singh, G. K., Daus, G. P., Allender, M., Ramey, C. T., Martin, E. K., Perry, C., Reyes, A. A. L., & Vedamuthu, I. P. (2017). Social determinants of health in the United States: Addressing major health inequality trends for the nation, 1935–2016. *International Journal of MCH and AIDS*, *6*(2), 139–164. 10.21106/ijma.236 PMC5777389 29367890

[9] McCartney, G., Dickie, E., Escobar, O., & Collins, C. (2021). Health inequalities, fundamental causes and power: Towards the practice of good theory. *Sociology of Health & Illness*, *43*(1), 20–39. 10.1111/1467-9566.13181 PMC7894306 33222244

[10] Michener, J. (2022). Health justice through the lens of power. *Journal of Law Medicine & Ethics*, *50*(4), 656–662. 10.1017/jme.2023.5 PMC10009378 36883395

[11] Reynolds, M. M., & Galea, S. (2025). Advancing the study of power: Opportunities and priorities for understanding population health inequities. *American Journal of Public Health*, *115*(6), 883–889. 10.2105/ajph.2025.308015 PMC12080447 40179346

[12] Voltz, R., Meesters, S., Ohler, K., Weihrauch, B., Kreische, A., Niessen, J., Heller, A., Strupp, J., & Kremeike, K. (2024). Top-down and bottom-up or participation through action? How to build a compassionate community - the experience of Caring Community Cologne. *Palliative Care and Social Practice*, *18*, 26323524241238230. 10.1177/26323524241238230 PMC10962028 38529387

[13] Centers for Disease Control and Prevention (2024, May 15). *Social determinants of health*. CDC. https://www.cdc.gov/public-health-gateway/php/about/social-determinants-of-health.html

[14] Dawes, D. E., Amador, C. M., & Dunlap, N. J. (2022). The Political Determinants of Health: A global panacea for health inequities. *Oxford Research Encyclopedia of Global Public Health*. 10.1093/acrefore/9780190632366.013.466

[15] Pastor, M., Speer, P., Gupta, J., Han, H., & Ito, J. (2022). Community power and health equity: Closing the gap between scholarship and practice. *NAM Perspectives*, *2022*. 10.31478/202206c. PMC9499379 36177207

[16] Dubowitz, T., Nelson, C., Weilant, S., Sloan, J., Bogart, A., Miller, C., & Chandra, A. (2020). Factors related to health civic engagement: Results from the 2018 National Survey of Health Attitudes to understand progress towards a Culture of Health. *Bmc Public Health*, *20*, 635. 10.1186/s12889-020-08507-w PMC7203885 32380964

[17] O’Mara-Eves, A., Brunton, G., McDaid, D., Oliver, S., Kavanagh, J., Jamal, F., Matosevic, T., Harden, A., & Thomas, J. (2013). Community engagement to reduce inequalities in health: A systematic review, meta-analysis and economic analysis. *Public Health Research*, *1*(4), 1–526. 10.3310/phr01040 25642563

[18] Habib, D., Klein, L. M., Perrin, E. M., Perrin, A. J., & Johnson, S. B. (2023). The role of primary care in advancing civic engagement and health equity: A conceptual framework. *Milbank Quarterly*, *101*(3), 731–767. 10.1111/1468-0009.12661 PMC10509514 37347445

[19] Lukes, S. (1974). *Power: A Radical View*. Macmillan.

[20] Veneklasen, L., & Miller, V. (2002). *A new weave of people, power and politics: The action guide for advocacy and citizen participation*. World Neighbors.

[21] Heller, J. C., Little, O. M., Faust, V., Tran, P., Givens, M. L., Ayers, J., & Farhang, L. (2023). Theory in action: Public health and community power building for health equity. *Journal of Public Health Management and Practice*, **29**(1), 33–38. 10.1097/phh.0000000000001681 36448756

[22] Carman, K. L., & Workman, T. A. (2017). Engaging patients and consumers in research evidence: Applying the conceptual model of patient and family engagement. *Patient Education and Counseling*, *100*(1), 25–29. 10.1016/j.pec.2016.07.009 27473639

[23] Haldane, V., Chuah, F. L. H., Srivastava, A., Singh, S. R., Koh, G. C. H., Seng, C. K., & Legido-Quigley, H. (2019). Community participation in health services development, implementation, and evaluation: A systematic review of empowerment, health, community, and process outcomes. *Plos One*, *14*(5), e0216112. 10.1371/journal.pone.0216112 PMC6510456 31075120

[24] Morales-Garzón, S., Parker, L. A., Hernández-Aguado, I., González-Moro Tolosana, M., Pastor-Valero, M., & Chilet-Rosell, E. (2023). Addressing health disparities through community participation: A scoping review of co-creation in public health. *Healthcare*, *11*(7), 1034. 10.3390/healthcare11071034 PMC10094395 37046961

[25] Organizing Committee for Assessing Meaningful Community Engagement in Health & Health Care Programs & Policies. (2022). Assessing meaningful community engagement: A conceptual model to advance health equity through transformed systems for health. *NAM Perspectives*. 10.31478/202202c PMC9303007 35891775

[26] Plamondon, K., Banner, D., Cary, M. A., Faulkner, M., Gainforth, H., Med, K. G., Hoens, A., Huisken, A., Kandola, D. K., Khan, S., Silva, A. S., Oelke, N., Rai, A., Strain, K., Sibley, K. M., & Wick, U. (2023). Relational practices for meaningful inclusion in health research: Results of a deliberative dialogue study. *Health Expectations*, *27*(1), e13865. 10.1111/hex.13865 PMC10726058 37749963

[27] Ravaghi, H., Guisset, A. L., Elfeky, S., Nasir, N., Khani, S., Ahmadnezhad, E., & Abdi, Z. (2023). A scoping review of community health needs and assets assessment: Concepts, rationale, tools and uses. *BMC Health Services Research*, *23*(1), 1–20. 10.1186/s12913-022-08983-3 PMC9847055 36650529

[28] den Broeder, L., Uiters, E., ten Have, W., Wagemakers, A., & Schuit, A. J. (2017). Community participation in Health Impact Assessment. A scoping review of the literature. *Environmental Impact Assessment Review*, *66*, 33–42. 10.1016/j.eiar.2017.06.004

[29] Carlin, T. (2022, October 27). Greenville County organizations receive $1.5 million grant to address health inequities. *Greenville News*. https://www.greenvilleonline.com/story/news/local/2022/10/27/greenville-sc-groups-get-1-5-million-address-health-inequities/69590310007/

[30] U.S. Census Bureau (2021). *QuickFacts: Greenville County, South Carolina*. U.S. Department of Commerce. Retrieved July 15, 2022, from https://www.census.gov/quickfacts/fact/table/greenvillecountysouthcarolina/PST045222

[31] Chetty, R., Jackson, M. O., Kuchler, T., Stroebel, J., Hendren, N., Fluegge, R. B., Gong, S., Gonzalez, F., Grondin, A., Jacob, M., Johnston, D., Koenen, M., Laguna-Muggenburg, E., Mudekereza, F., Rutter, T., Thor, N., Townsend, W., Zhang, R., Bailey, M., & Barberá, P. (2022). Social capital I: Measurement and associations with economic mobility. *Nature*, *608*, 108–121. 10.1038/s41586-022-04996-4 PMC9352590 35915342

[32] Clarke, V., & Braun, V. (2017). Thematic analysis. *The Journal of Positive Psychology*, *12*(3), 297–298. 10.1080/17439760.2016.1262613

[33] House-Niamke, S., & Eckerd, A. (2020). Institutional injustice: How public administration has fostered and can ameliorate racial disparities. *Administration & Society*, *53*(2). 10.1177/0095399720979182

[34] Mittal, P., & Bansal, R. (2024). *Community Engagement for Sustainable Practices in Higher Education*. Palgrave Macmillan. 10.1007/978-3-031-63981-4_13

[35] Garcia, A. A., Ohueri, W., Garay, C., Guzmán, R., Hanson, M., Vasquez, K., Zuñiga, M., J., & Tierney, W. (2021). Community Engagement as a Foundation for Improving Neighborhood Health. *Public Health Nursing*, *38*(2), 223–231. 10.1111/phn.12870 33522011

[36] Deal, H. E. (2022). Christian congregations and social and political action: A review of the literature. *Social Work and Christianity*, *49*(3), 289–301. 10.34043/swc.v49i3.303

[37] Lindsay, D. M. (2007). Ties that bind and divisions that persist: Evangelical faith and the political spectrum. *American Quarterly*, *59*(3), 883–909. 10.2307/40068454

[38] Steensland, B., & Wright, E. L. (2014). American evangelicals and conservative politics: Past, present, and future. *Sociology Compass*, *8*(6), 705–717. 10.1111/soc4.12175

[39] Wood, R. L., & Warren, M. R. (2002). A different face of faith-based politics: Social capital and community organizing in the public arena. *International Journal of Sociology and Social Policy*, *22*(11/12), 6–54. 10.1108/01443330210790148

[40] Schneider, J. A. (2016). Envisioning religiously diverse partnership systems among government, faith communities and fbos. *Religions*, *7*(8), 105. 10.3390/rel7080105

[41] Christens, B. D. (2019). *Community power and empowerment*. Oxford University Press.

[42] Butt, B. (2023). Unveiling the invisible: Power, inequality, and the dynamics of social stratification. *Research Journal of Psychology*, *1*(2), 84–90. 10.59075/rjs.v1i2.13

[43] Wang, X., Pei, T., Ci, S., Chen, J., Shu, H., Liu, Y., Guo, S., & Chen, X. (2023). How does socioeconomic status influence social relations? A perspective from mobile phone data. *Physica A: Statistical Mechanics and Its Applications*, *615*, 128612. 10.1016/j.physa.2023.128612

